# Molecular Dissection of dH3w, A Fluorescent Peptidyl Sensor for Zinc and Mercury

**DOI:** 10.3390/s20030598

**Published:** 2020-01-21

**Authors:** Marialuisa Siepi, Rosario Oliva, Filomena Battista, Luigi Petraccone, Pompea Del Vecchio, Viviana Izzo, Fabrizio Dal Piaz, Rachele Isticato, Eugenio Notomista, Giuliana Donadio

**Affiliations:** 1 Department of Biology, University of Naples Federico II, Via Cintia, 80126 Naples, Italy; marialuisa.siepi@unina.it (M.S.); rachele.isticato@unina.it (R.I.); 2 Physical Chemistry I, TU Dortmund University, Otto-Hahn-Str. 4a, 44227 Dortmund, Germany; rosario.oliva2@unina.it; 3 Department of Chemical Sciences, University of Naples Federico II, Via Cintia, 80126 Naples, Italy; filomena.battista@unina.it (F.B.); luigi.petraccone@unina.it (L.P.); pompea.delvecchio@unina.it (P.D.V.); 4 Department of Medicine, Surgery and Dentistry “Scuola Medica Salernitana”, University of Salerno, Via Salvador Allende, 84081 Baronissi, Italy; vizzo@unisa.it (V.I.); fdalpiaz@unisa.it (F.D.P.)

**Keywords:** fluorescent sensor, peptidyl sensor, metal binding peptide, zinc detection, mercury detection

## Abstract

Previously, we reported that fluorescent peptide dansyl-HPHGHW-NH_2_ (dH3w), designed on the repeats of the human histidine-rich glycoprotein, shows a turn-on response to Zn(II) and a complex response to Hg(II) characterized by a turn-off phase at low Hg(II) concentrations and a turn-on phase at high concentrations. As Hg(II) easily displaces Zn(II), dH3w is a useful probe for the environmental monitoring of Hg(II). In order to investigate the molecular basis of the metal selectivity and fluorescence response, we characterized three variants, dH3w(H1A), dH3w(H3A), and dH3w(H5A), in which each of the three histidine residues was changed to alanine, and two variants with a single fluorescent moiety, namely dH3w(W6A), in which the tryptophan residue at the C-terminus was changed to alanine, and AcH3w, in which the N-terminal dansyl moiety was substituted by an acetyl group. These variants allowed us to demonstrate that all the histidine residues are essential for a strong interaction with Zn(II), whereas two histidine residues (in particular His5) and the dansyl group are necessary to bind Hg(II). The data reported herein shed light on the molecular behavior of dH3w, thus paving the way to the rational designing of further and more efficient fluorescent peptidyl probes for Hg(II).

## 1. Introduction

Heavy metal pollution is of great concern due to the toxicity and the impact on natural ecosystems of several heavy metals [1,2]. Mercury is particularly worrying because it is one of the most toxic metals and easily accumulates in the food chain, a phenomenon called biomagnification. This raises the need for a continuous environmental monitoring of heavy metals, in general, and of mercury, in particular. Fluorescent chemical sensors are very well suited for this task as they are relatively easy to prepare and fluorescence detection does not require expensive or bulky instruments [3]. The ability of thiols to react with mercury ions is used for the creation of mercury-sensing luminescent nanomaterials [4]. However, the synthesis of these materials is often complicated, and their fluorescence depends on agglomeration, which, in turn, might be mercury-induced or material concentration-induced that requires a careful analysis of the results [5]. In contrast, fluorescent peptidyl sensors are very interesting as they can be easily prepared by standard solid-phase synthesis and their chemical-physical properties can be finely tuned by changing the amino acid sequence [6]. Moreover, many natural metal-binding proteins can be used as a convenient source of small peptides or amino acid motifs with defined metal-binding specificities.

Previously, we described a dansylated peptidyl sensor, dansyl-HPHGHW-NH_2_ (dH3w), designed on the basis of the internal repeats of human histidine-rich glycoprotein (HPHGH) [7,8]. dH3w shows a turn-on response to Zn(II), a turn-off response to Cu(II), and a complex response to Hg(II), characterized by a turn-off phase at low Hg(II) concentrations and a turn-on phase at high concentrations. Moreover, dH3w has an affinity for Hg(II) 1000 times higher than that for Zn(II); therefore, Hg(II) can displace Zn(II) completely from the peptide, thus causing a strong turn-off response of the Zn(II)/dH3w complex.

Interestingly, dH3w shows very different binding modes for Zn(II) and Hg(II). In the presence of Zn(II), dH3w forms a dimeric complex with a stoichiometry 2:1, peptide/zinc [7]. Likely, the dimerization of the peptide causes the turn-on response by changing the exposure to the solvent of the dansyl group, a well-known solvatochromic fluorophore [9]. This explanation is also supported by a blue shift from 560 to 515 nm in the emission upon binding/dimerization. On the contrary, dH3w forms at least two complexes with Hg(II) with stoichiometries 1:1 and 1:2, peptide/mercury [8]. We hypothesized that the formation of the 1:1 complex at low Hg(II) concentration is responsible for the turn-off response, whereas the formation of the 1:2 complex at high Hg(II) concentration is responsible for the turn-on response. We also hypothesized that the turn-off response would be caused by mercury binding to the imidazole moieties of the histidine residues, whereas the turn-on response, accompanied by a strong blue shift from 560 to 510 nm, would be caused by the direct binding of dansyl sulfonamide to mercury [8,10]. Unfortunately, all our attempts to determine the NMR structures of the dH3w/Zn(II) and dH3w/Hg(II) complexes were unfruitful. This prevented the possibility to directly verify the binding modes of zinc and mercury ions.

In order to study the binding process, we designed four variants of dH3w, each lacking one of the four groups, which could likely work as metal ligands, that is, the imidazole moieties of the histidine residues at positions 1, 3, and 5 and the sulfonamide moiety of the dansyl group at the N-terminus. To this aim, the histidine residues were changed to alanine, to obtain the variants called dH3w(H1A), dH3w(H3A), and dH3w(H5A), whereas the dansyl group was changed to an acetyl group in the variant AcH3w. Moreover, we also prepared a variant, dH3w(W6A), lacking the indole moiety at the C-terminus, in order to study the influence of the tryptophan residue on the fluorescence. The characterization of the fluorescence of the five variants provided information on the role in metal binding and fluorescence modulation of the three histidine residues, the dansyl group, and the tryptophan residue.

## 2. Materials and Methods

### 2.1. Materials

All the peptides used were synthesized by Primm s.r.l. (Milan, Italy) with a purity grade of 98%. Stock solutions of metal ions (zinc or mercury) were prepared by dissolving the appropriate chloride salt in 20 mM 3-(N-morpholino)propanesulfonic acid (MOPS) buffer, pH 7.0. All sample solutions were prepared by appropriate dilution of stock solutions. All chemicals were of reagent grade and used without further purification. All the solutions were prepared using deionized distilled water.

### 2.2. Steady-State Fluorescence Spectroscopy

Fluorescence experiments were performed with a Fluoromax-4 from Horiba Scientific (Edison, NJ, USA) using a 1 cm path length quartz cuvette. The temperature was set at 25 °C and controlled with a Peltier system that ensured an accuracy of ±0.1 °C. The excitation wavelength was set at 340 nm for dansyl-labeled peptides. In the case of AcH3w, the fluorescence spectra were obtained after excitation of the tryptophan residue at 295 nm. Both excitation and emission slit were set at 10 nm. The titrations were performed by recording the emission spectra of peptides at a fixed concentration of 7 μM and varying the concentration of zinc from 0 to 1.6 mM and mercury from 0 to 800 µM. All the experiments were performed in 20 mM MOPS buffer, pH 7.0. The estimation of the binding constant (where possible) was performed by plotting the fraction of bound peptide (α) as function of total metal concentration, and fitting the experimental data according to an equivalent and independent binding site model, as described in detail in [7].

### 2.3. Monte Carlo Modeling of Zn(dH3w)_2_

The structures of eight octahedral and eight tetrahedral Zn(dH3w)_2_ complexes with all the possible protonation states of His1, His3, and His5 were modelled using a Monte Carlo energy minimization strategy that has already proved useful for the modeling of several complexes of different nature and size [11,12,13,14,15,16,17]. All calculations were performed using the ZMM-MVM molecular modeling package (ZMM Software Inc., 2011, Dundas, ON, Canada, http://www.zmmsoft.com). ZMM software allows conformational searches using generalized coordinates instead of Cartesian coordinates [18], thus making the conformational search faster than with other methods. Atom–atom interactions were evaluated using the Amber force fields [19], with a cutoff distance of 8 Å. Conformational energy calculations included van der Waals, electrostatic, H bond, and torsion components. Electrostatic interactions were calculated using an environment- and distance-dependent dielectric permittivity according to a method implemented in the ZMM software. Energy calculations also included a hydration component [19]. Atom-to-atom distance constrains were used to fix the distances of the desired Nδ and Nε nitrogen atoms of His1, His3, and His5 from the zinc ion (distance was constrained between 1.9 and 2.2 Å). A twofold symmetry axis was imposed in all the calculations. Three initial structures were prepared for each complex using PyMOL (DeLano Scientific LLC, version 0.99-rc6, Palo Alto, CA, USA), https://www.pymol.org/) and used as starting points for all the Monte Carlo trajectories. Trajectories were stopped when no energy decrease was observed for 1000 minimization cycles.

## 3. Results and Discussion

### 3.1. Response to Zn(II) of the dH3w Variants

**Figure 1:**
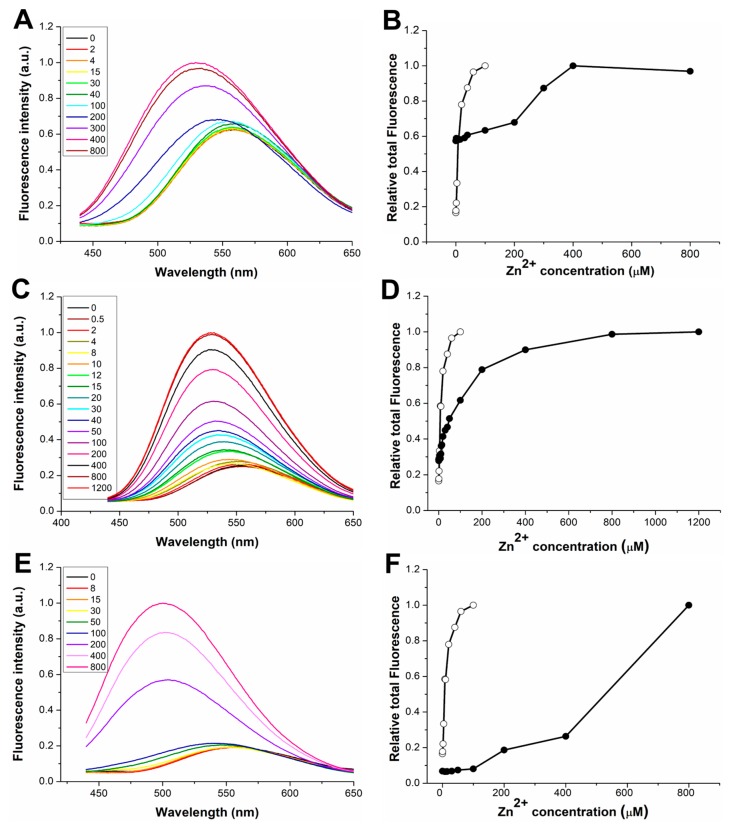
Fluorescence response of dH3w(H1A), dH3w(H3A), and dH3w(H5A) as function of Zn(II) concentration. Emission spectra of (**A**) dH3w(H1A), (**C**) dH3w(H3A), and (**E**) dH3w(H5A). The legends show the concentrations of Zn(II) in µM units. Normalized area of the fluorescence emission as function of the Zn(II) concentration for (**B**) dH3w(H1A), (**D**) dH3w(H3A), and (**F**) dH3w(H5A) (filled circles), respectively. For comparison, the normalized area of the fluorescence emission of dansyl-HPHGHW-NH_2_ (dH3w) is also reported in each panel (void circles). Spectra were registered after excitation at 340 nm (the absorption maximum of the dansyl moiety) in 20 mM 3-(N-morpholino)propanesulfonic acid (MOPS) buffer, pH 7 at 25 °C.

Figure 1 shows the fluorescence emission spectra of dH3w(H1A), dH3w(H3A), and dH3w(H5A) at increasing concentrations of zinc ions in 20 mM MOPS buffer, pH 7 after excitation at 340 nm (the absorption maximum of the dansyl moiety). The panels on the right show the integration of the spectra as function of zinc concentration.

Even if all the variants show a turn-on response to Zn(II), the details of this response are different among the variants and with respect to the parent peptide. The increase in fluorescence intensity is considerably lower in the dH3w(H1A) variant (1.7-fold) than in dH3w(H3A) and dH3w(H5A) (3.6- and 5.2-fold, respectively). Upon Zn(II) binding to dH3w(H1A) and dH3w(H3A), the λ_max_ values shift from about 560 nm to about 530 nm (Table 1), a blue shift considerably lower than that observed in the case of the parent peptide (from 560 to 515 nm). On the contrary, in the case of dH3w(H5A), the λ_max_ value shifts from 560 nm to about 500 nm. The turn-on response and the blue shift in the λ_max_ value have been observed in several other dansylated peptidyl sensors that, like dH3w, form zinc complexes with stoichiometry 2:1, peptide/metal. The most likely explanation is that both changes depend on the well-known solvatochromic nature of the dansyl fluorophore, which determines an increase in the fluorescence intensity and a blue shift in the λ_max_ value when the fluorophore moves from a more polar to a less polar environment [9]. The zinc-induced dimerization causes a reduction in the exposure of the dansyl group to the solvent, thus changing the emission spectrum. Additionally, chelation-enhanced fluorescence (CHEF) may contribute to increase fluorescence intensity, but CHEF does not provide an explanation for the blue shift in the λ_max_ values.

On this background, the differences in the fluorescence of dH3w(H1A), dH3w(H3A), and dH3w(H5A) in the presence of Zn(II) would indicate that, although all the peptides are able to bind Zn(II) (presumably in a dimeric form), the structures of the three complexes are slightly different from that of the complex dH3w/Zn(II). The plots of the integration of the spectra as function of zinc concentration clearly show that dH3w(H3A) has a behavior more similar to that of the parent peptide than to those of dH3w(H1A) and dH3w(H5A). In fact, only in the case of dH3w(H3A) does the plot show the expected hyperbolic shape with a fluorescence change already appreciable at 8–10 μM of zinc. The plots of dH3w(H1A) and dH3w(H5A), on the other hand, clearly reveal a weaker dependence of fluorescence emission on the zinc concentration with a significant increase of fluorescence intensity only observable at zinc concentrations higher than 100–200 μM.

**Figure 2:**
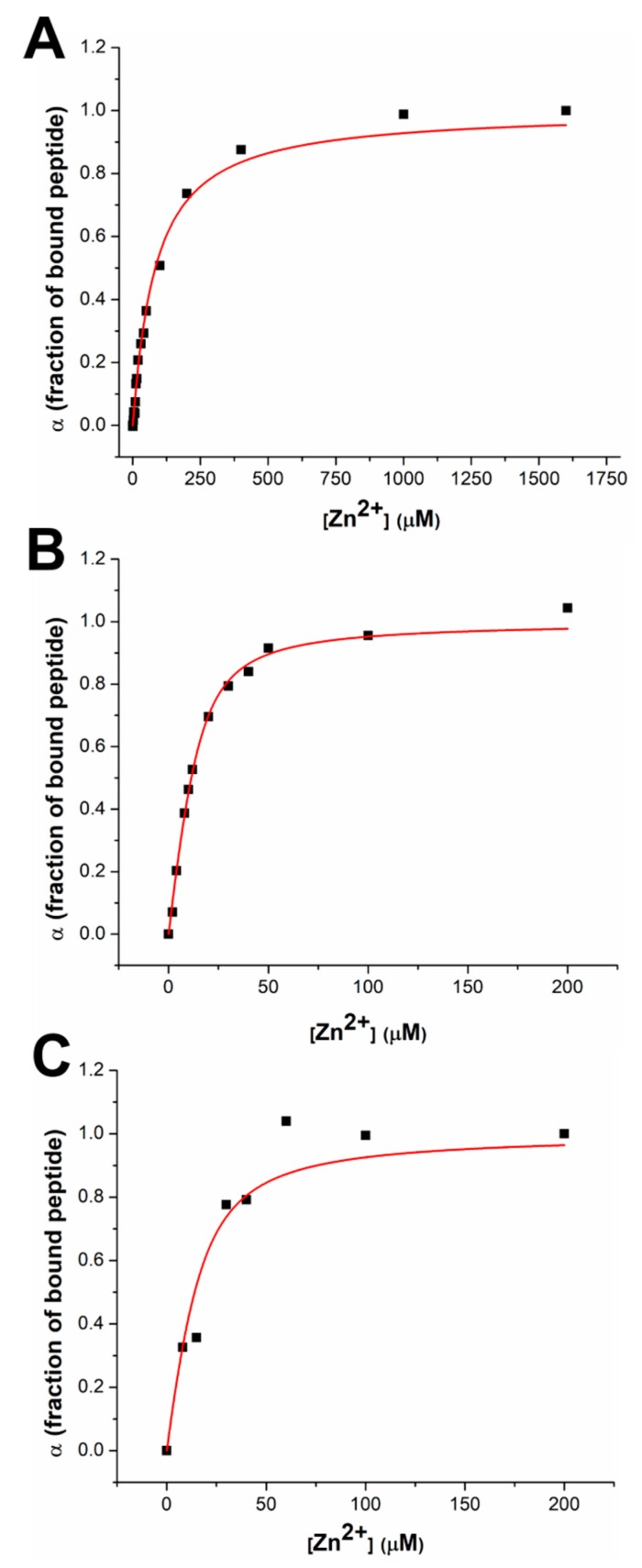
Zn(II) binding curves for peptides (**A**) dH3w(H3A), (**B**) dH3w(W6A), and (**C**) AcH3w. Binding curves were obtained by plotting the fraction of bound peptide (α) versus the Zn(II) concentration. K_b_ values and stoichiometries derived from the fitting procedure are shown in Table 2.

By plotting the fraction of bound dH3w(H3A) (α) versus the Zn(II) concentration, it was possible to quantitatively estimate the binding constant for the interaction of dH3w(H3A) with the metal ion (Figure 2). The value of the binding constant found for dH3w(H3A), 2.6 × 10^4^ M^−1^, is twenty times lower than the value of the binding constant measured for dH3w (Table 2). Unfortunately, in the case of dH3w(H1A) and dH3w(H5A), the fluorescence response was too complex to determine the values of the binding constants. Nevertheless, a qualitative estimation of the binding constants is possible. In fact, taking into account only the Zn(II) concentrations where a fluorescence change is detectable, a tentative value of the binding constant for H1A and H5A could be around 10^3^ × M^−1^. It can be reasonably concluded that dH3w(H1A) and dH3w(H5A) have an affinity for Zn(II) lower than that of dH3w(H3A). 

**Figure 3:**
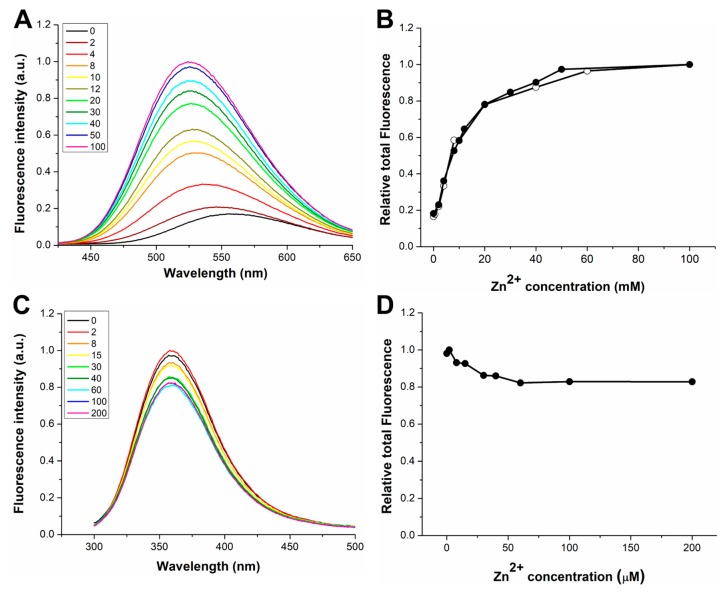
Fluorescence response of dH3w(W6A) and AcH3w as function of Zn(II) concentration. Emission spectra of (**A**) dH3w(W6A) and (**C**) AcH3w. The legends show the concentrations of Zn(II) in µM units. Normalized area of the fluorescence emission as function of the Zn(II) concentration for (**B**) dH3w(W6A) and (**D**) AcH3w (filled circles). (**B**) The normalized area of the fluorescence emission of dH3w (void circles) is also shown for comparison. Spectra of dH3w(W6A) were registered after excitation at 340 nm (the absorption maximum of the dansyl group). Spectra of AcH3w were registered after excitation at 295 nm.

We also characterized the interaction with Zn(II) in the case of dH3w(W6A) and AcH3w (Figure 3). Even if these variants were designed mainly to investigate the interaction of dH3w with Hg(II), the characterization of their complexes with zinc nonetheless provided interesting indications. In the presence of Zn(II), dH3w(W6A) showed a behavior essentially identical to that of dH3w, except that the λ_max_ value is red shifted by about 10 nm, thus indicating that the tryptophan residue is, at least in part, responsible for the shielding of the dansyl group from the solvent upon the formation of the dimeric complex (Figure 3A,B). Interestingly, AcH3w showed an unexpected albeit weak turn-off effect given the fact that Zn(II) is a redox-inactive metal usually unable to induce fluorescence quenching (Figure 3C,D). The observed small reduction in the fluorescence emission could be the result of the dimerization of the peptide, which could determine the quenching of the tryptophan residues by internal quenchers [20,21]. By plotting the fraction of bound peptide (α) versus the Zn(II) concentration (Figure 2B,C), it was possible to determine the binding constant of dH3w(W6A) and AcH3w and the stoichiometry (only for dH3w(W6A)), which, as expected, are very similar to those found for dH3w (Table 2).

### 3.2. Modeling of the dH3W/Zn(II) Complex

Overall, the data reported in the previous section clearly demonstrate that all the histidine residues in dH3w are essential for a high-affinity binding of Zn(II). In fact, the removal of a single imidazole group causes a reduction in the affinity constant of at least two orders of magnitude. It is also clear that changing His3 to alanine is somewhat less deleterious than changing His1 or His5. The schematic structures of the dimeric zinc/peptide complexes shown in Figure S1 (Supplementary Materials) offer a possible explanation to the observed differences among the dH3w variants. The figure shows two alternative structures for the dH3w/Zn(II) complex—an octahedral complex, in which each of the three histidine residues is directly coordinated to zinc, and a tetrahedral complex, in which only His1 and His5 are directly bound to zinc, whereas His3 is part of a turn (Pro2–His3–Gly4) holding in position the coordinating histidines. In biological complexes, zinc is frequently tetra-coordinated with a tetrahedral geometry (zinc fingers are classical examples); nonetheless, several zinc complexes with octahedral geometry are known, especially when all the ligands are nitrogen atoms, like in the hexakis(imidazole)zinc(II) complex [22] or in the complexes of zinc with the unsubstituted tris(pyrazolyl)borate anion [23] and other similar tripodal ligands [24]. Therefore, even if the tetrahedral complex is slightly more likely, we cannot exclude the possibility that dH3w forms a complex with octahedral geometry. It is worth noting that the reduction in the affinity constant by two orders of magnitude, observed in the case of dH3w(H3A), may be due either to the loss of a ligand in the octahedral complex or to the destabilization of the turn-like structure necessary for the correct positioning of His1 and His5 in the tetrahedral complex. Therefore, the observed reduced affinity of dH3w(H3A) for zinc is not sufficient to choose between the two alternative geometries. On the other hand, changing His1 or His5 inevitably determines a change in the coordination mode, either causing the loss of one of the ligands (in the hypothesis of an octahedral complex) or changing the spacing of the coordinating histidines from a H(X)_3_H to a HXH (in the hypothesis of a tetrahedral complex).

**Figure 4:**
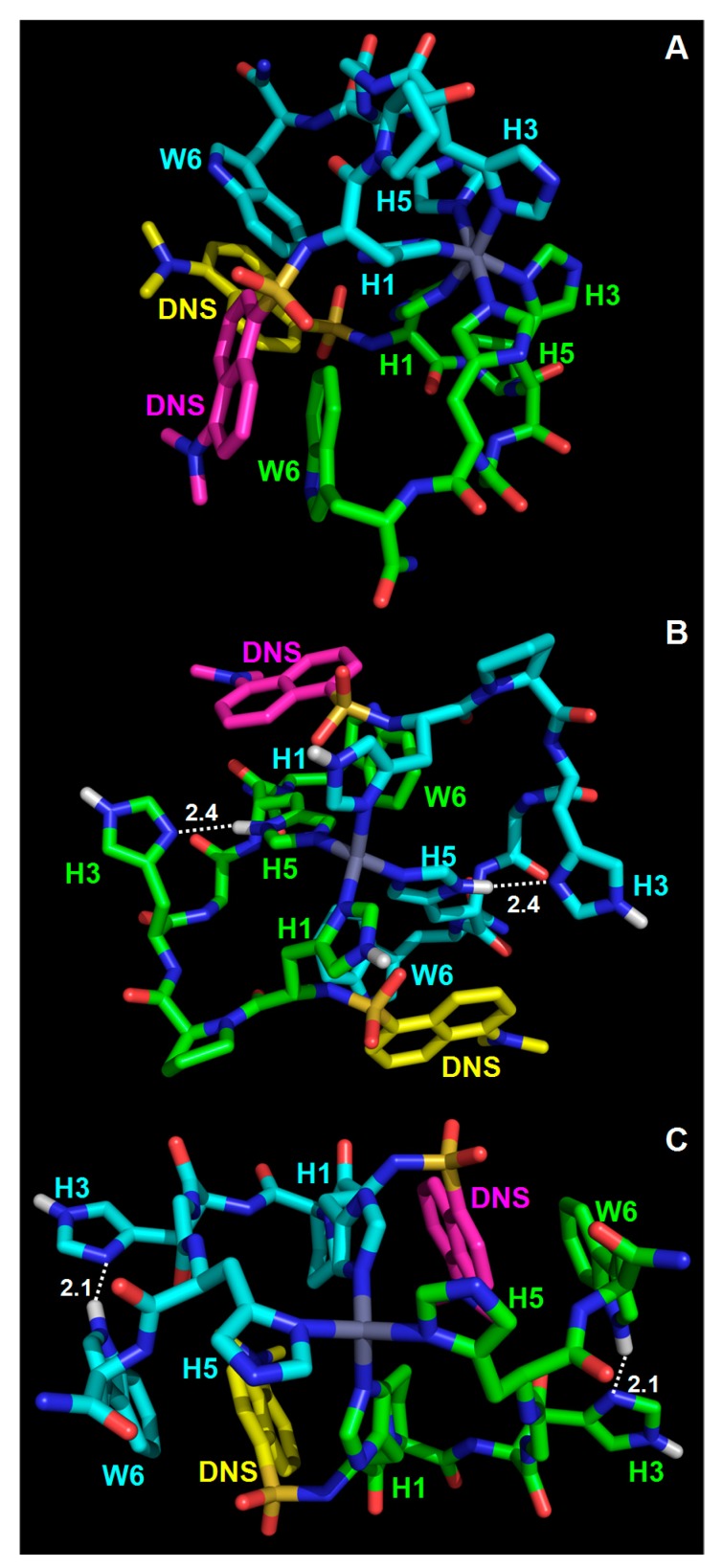
Representative Monte Carlo minimized models of the Zn(dH3w)_2_ complex. (**A**) The most stable octahedral complex model. His1, His3, and His5 are coordinated through the Nε, Nδ, and Nε nitrogen atoms, respectively. (**B**) Tetrahedral complex model in which His1 and His5 are coordinated through the Nδ and Nε nitrogen atoms, respectively, whereas His3 is protonated at the Nε nitrogen atom and, with its Nδ nitrogen atom, is H-bonded to the H-Nδ group of His5. (**C**) Tetrahedral complex model in which His1 and His5 are coordinated through the Nε and Nδ nitrogen atoms, respectively, whereas His3 is protonated at the Nε nitrogen atom and, with its Nδ nitrogen atom, is H-bonded to the H–N group of the indole moiety of Trp6. Residues are shown as sticks colored by atom type: nitrogen, blue; oxygen, red; sulphur, dark yellow. Carbon atoms of the two peptide molecules are green and cyan, except for the carbon atoms of the dansyl groups (DNS) that are yellow and magenta, respectively. Zn(II) is gray. Hydrogen atoms are shown only when relevant and are white. Hydrogen bonds are shown as white dotted lines and the corresponding distances are in Å.

In order to obtain further insights on the metal-binding mode, we performed the in silico modeling of the (dH3w)_2_Zn(II) complex. This task is made particularly complex by the fact that each histidine residue could bind zinc either through the Nδ or the Nε nitrogen atoms. Therefore, an exhaustive search requires the modeling of eight different symmetric octahedral complexes, as listed in Table 3 (if non-symmetric complexes are considered, the number is obviously higher). Moreover, it should be remembered that a non-coordinated histidine can also bear the proton either at the Nδ or at the Nε atoms; therefore, the possible tetrahedral complexes are also eight (Table 3). We chose a Monte Carlo minimization strategy with implicit solvation [18,19,25] that, being computationally not demanding, is well suited to explore a large number of alternative complexes. The imidazole–Zn(II) interaction was arbitrarily considered purely ionic and distance constrains were used to define the desired Nδ–Zn(II) and Nε–Zn(II) interactions, whereas the geometry of the ligands around the metal center was not constrained. In spite of this choice, all the minimized complexes with six nitrogen atoms bound to the zinc ion showed a fairly regular octahedral geometry and all the minimized complexes with four nitrogen atoms bound to the zinc ion showed a tetrahedral geometry, slightly distorted in some complexes. Three representative examples are shown in Figure 4. This result is likely due to the fact that the tetrahedral and the octahedral arrangements minimize steric hindrance when four or six ligands, respectively, are bound to a metal ion. The energy values of the tetrahedral complexes were all similar with six values in the range of −1510 to −1527 kJ/mol, whereas in the case of the octahedral complexes two minimized models showed an energy significantly lower than those of the others (i.e., the models with His1 and His5 coordinated through their Nε nitrogen atoms). This is not unexpected given the flexibility of the peptide backbone and the fact that, from a steric point of view, the binding to the metal center of the two terminal histidine residues is likely much less demanding than the positioning of all the histidine residues.

The most stable octahedral model complex was about 125 kJ/mol less stable than the tetrahedral complexes. This difference was due mainly (about 92 kJ/mol) to the van der Waals energy component, thus confirming that crowding of the imidazole rings around the metal center is the main factor making the octahedral complexes less stable that the tetrahedral ones. On the other hand, it should be remembered that our analysis did not consider the covalent contribution of the imidazole–zinc bonds which could reduce, at least in part, the gap between the stability of octahedral and tetrahedral complexes. Very interestingly, the non-coordinating His3 in the tetrahedral complex models is often involved in interactions, stabilizing either the turn or the dimeric structure. For example, in the complex shown in Figure S2B (Supplementary Materials), His3 makes a H-bond with His5 from the same peptide, whereas, in the complex shown in Figure S2C His3 from each peptide unit makes a H-bond with Trp6 from the other peptide unit. Therefore, our analysis supports the hypothesis that His3 may have an important structural role even if it is not directly involved in zinc binding.

The models also show that, as a consequence of the dimerization process, the dansyl group becomes less exposed to the solvent. In most models, the dansyl group is closely packed with the tryptophan residue(s) as well as other residues both from the same peptide and from the second peptide in the dimer (three examples are shown in Figure S2). This is in agreement with the hypothesis that the turn-on response is the result of the solvatochromic nature of dansyl and with the finding that the removal of the indole moiety in dH3w(W6A) causes a significant reduction in the blue shift of the peptide/zinc complex (Table 1). Close packing in the dimers can also be the cause of the reduction in the fluorescence of tryptophan residues in the AcH3w variant. In fact, it is well known that tryptophan fluorescence can be quenched by internal groups in proteins including amide bonds and histidine residues [20,21].

Finally, for the sake of completeness, we also want to mention an alternative model including all the structures shown in Figure S1. As schematically shown in Figure S3 (Supplementary Materials), according to this model the (dH3w)_2_Zn(II) complex would be a fluxional molecule continuously changing structure, and in particular the octahedral complex would be an intermediate providing an easy path between the different tetrahedral complexes.

### 3.3. Response to Hg(II) of the dH3w Variants

**Figure 5:**
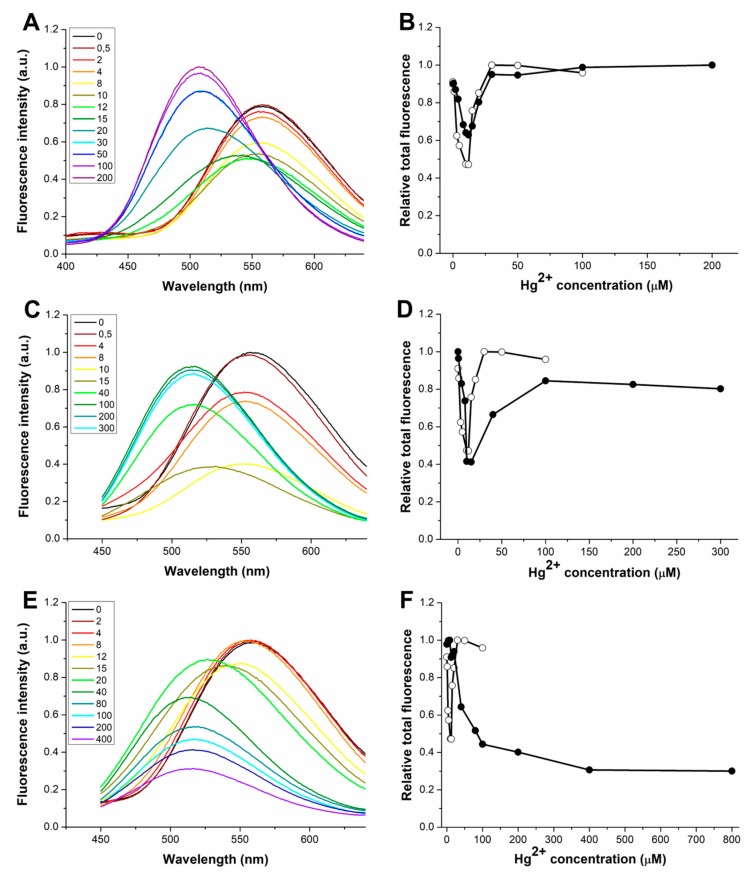
Fluorescence response of dH3w(H1A), dH3w(H3A), and dH3w(H5A) as function of Hg(II) concentration. Emission spectra of (**A**) dH3w(H1A), (**C**) dH3w(H3A), and (**E**) dH3w(H5A). The legends show the concentrations of Hg(II) in µM units. Normalized area of the fluorescence emission as function of the Hg(II) concentration for (**B**) dH3w(H1A), (**D**) dH3w(H3A), and (**F**) dH3w(H5A) (filled circles). For comparison, the normalized area of the fluorescence emission of dH3w is also reported in each panel (void circles). Spectra were registered after excitation at 340 nm (the absorption maximum of the dansyl group) in 20 mM MOPS buffer, pH 7 at 25 °C.

Figure 5 shows the fluorescence emission spectra of dH3w(H1A), dH3w(H3A), and dH3w(H3A) at increasing concentrations of Hg(II). Qualitatively, dH3w(H1A) and dH3w(H3A) showed the same general behavior of the parent peptide dH3w, that is, a turn-off response without a change in the λ_max_ at Hg(II) concentrations below 10 µM and a turn-on response at concentrations higher than 15 µM coupled with a strong blue shift in the λ_max_. In particular, reductions of 40% and 60% in the fluorescence intensities were observed at low Hg(II) for dH3w(H1A) and dH3w(H3A), respectively; these reductions were very similar to that previously measured for dH3w peptide (60%). Additionally, the integration of the spectra as function of Hg(II) concentration (Figure 5B,D and Figure S4A,B (Supplementary Materials)) highlights the similarities between the parent peptide and the two variants, and in particular the fact that the switch between the turn-off and the turn-on phase happens in the same Hg(II) concentration range (10–15 µM) for the three peptides. These data confirm that dH3w(H1A) and dH3w(H3A), like dH3w, should bind sequentially two Hg(II) ions and that the first binding event induces the quenching of the fluorescence, whereas the second one causes the turn-on response. The most relevant difference can be seen in the right part of the plot of dH3w(H3A) (Figure 5B), which increases more slowly than in the case of the parent peptide, thus suggesting that the binding event responsible for the turn-on phase in the case of dH3w(H3A) may be less favored than in the case of dH3w. On the other hand, in the case of dH3w(H1A), the part of the curve corresponding to the turn-off phase shows a lower slope than in the case of the parent peptide (Figure S4A). A quantitative analysis of the fluorescence data is not easy, due to the very complex variations observed. Nonetheless, qualitatively our data suggest that the H1A change slightly impairs the first binding event, whereas the H3A change slightly impairs the second binding event.

Conversely, in the case of dH3w(H5A), only a slight fluorescence change was detected up to 20 µM Hg(II), with a reduction of 10% in the fluorescence intensity and no change in the λ_max_ value, which was followed, at higher Hg(II) concentrations, by a decrease of the fluorescence intensity with a blue shift up to 510 (Figure 5E,F and Figure S4C). This clearly demonstrates that His5 has an important role in the binding of Hg(II) at low metal concentration and it is essential for the turn-on response at higher Hg(II) concentrations.

**Figure 6:**
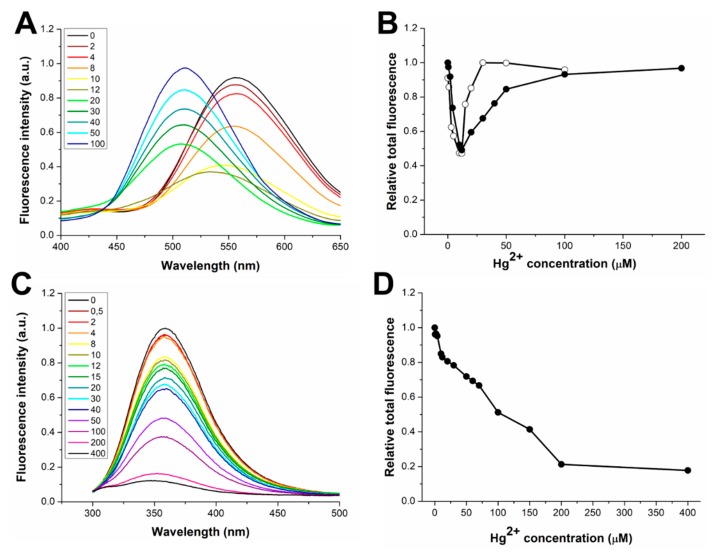
Fluorescence response of dH3w(W6A) and AcH3w as function of Hg(II) concentration. Emission spectra of (**A**) dH3w(W6A) and (**C**) AcH3w. The legends show the concentrations of Hg(II) in µM units. Normalized area of the fluorescence emission as function of the Hg(II) concentration for (**B**) dH3w(W6A) and (**D**) AcH3w (filled circles). (**B**) The normalized area of the fluorescence emission of dH3w (void circles) is also shown for comparison. Spectra of dH3w(W6A) were registered after excitation at 340 nm (the absorption maximum of the dansyl group). Spectra of AcH3w were registered after excitation at 295 nm.

Furthermore, dH3(W6A) behaves similarly to the parent peptide dH3w—a decrease of fluorescence intensity was observed in the concentration range 0–12 µM (Figure 6A,B) followed by an increase of intensity coupled with a strong blue shift of the λ_max_ value in the range 12–200 µM. However, as in the case of dH3w(H3A), the loss of the indole group apparently slightly impairs the binding event responsible for the turn-on phase (Figure 6B and Figure S5A (Supplementary Materials)).

Finally, in the case of AcH3w (which lacks the dansyl group), and upon increasing the mercury concentration, we detected the expected progressive and strong decrease of the tryptophan fluorescence (Figure 6C,D). This confirms that the turn-on phase is strictly dependent on the presence of the dansyl group. However, a close inspection of the plot of the total fluorescence emission at low Hg(II) concentrations (Figure S5B) shows two linear variations with different slopes, thus suggesting the existence, also in this case, of at least two different binding events.

### 3.4. Modeling of the Hg/dH3w Complexes

As a high degree of covalence is typical of Hg(II) complexes, we did not attempt to perform the Monte Carlo minimization of the Hg/dH3w complexes. Nonetheless, on the basis of literature data and of the analysis reported in the previous section, it is possible to make reliable hypotheses on their structures. Due to electronic reasons, Hg(II) has a strong propensity to linear bicoordination [26]. Only in the presence of a large excess of ligand or inside protein complexes can a third ligand bind to Hg(II), but even in those cases the three ligands are not necessarily equivalent. An interesting example is given by a family of cyclic peptides with three cysteine residues (arranged in the motif CXCXXC), which show several similarities to dH3w [27]. These peptides form 1:1 (peptide/metal) complexes at low mercury concentrations and, at higher concentrations, 1:2 and 2:3 (peptide/metal) complexes. In the 1:1 (peptide/metal) complex, Hg(II) is linearly coordinated, at acidic pH, and tricoordinated, at neutral pH, but with a T-shaped geometry characterized by two equivalent short bonds and a longer (weaker) one. In the 1:2 and 2:3 (peptide/metal) complexes, the authors hypothesized that the first Hg(II) is coordinated by two cysteine residues from the same peptide molecule, whereas the second Hg(II) is bound to the third cysteine residue and to a chloride ion (in the 1:2 complex) or a second peptide molecule (in the 2:3 complex). In analogy to these cyclic peptides and on the basis of our experimental data, we hypothesized the most likely structures of the Hg complexes of dH3w and of the variants with two histidine residues (Figure S6, Supplementary Materials). On the basis of the behavior of dH3w(H1A), dH3w(H3A), and dH3w(H5A) at low Hg(II) concentrations (Figure S4), we can hypothesize the following order of relative affinity for Hg(II) of the histidine motifs in dH3w: HPXGH ≥ HGH >> HPH. Accordingly, the first Hg(II) ion would be bound by His1 and His5 or His3 and His5, likely with similar affinities even if the complex involving His1 and His5 may be slightly more stable as evidenced by the behavior of dH3w(H1A) at low Hg(II) concentrations as described in the previous section. The complex with mercury bound to His1 and His3 (HPH motif), likely, gives a minor contribution to the binding as dH3w(H5A) binds Hg(II) only at high concentrations. At high concentrations of Hg(II), as we have previously suggested [8], a second metal ion would bind directly to the sulfonamide moiety of the dansyl group, thus causing the turn-on phase. As the characterization of the peptides dH3w(H1A), dH3w(H3A), and dH3w(H5A) clearly demonstrates that only His5 is essential for the formation of the highly fluorescent complex, it can be confidently hypothesized that, in this complex, one of the mercury ions is bound between the sulfonamide and His5, whereas the other is bound to His1, His3, or both. This model is in agreement with the finding that the behavior of dH3w(H1A) and dH3w(H3A) at high Hg(II) concentrations is very similar to that of dH3w. It is also worth noting that, according to this model, the turn structure performs an important role in Hg(II) binding similarly to what is found in the case of zinc binding. 

In the case of dH3w(H5A), a complex can be detected only at high Hg concentrations, likely due to the lower affinity for Hg(II) of the HPH motif with respect to the HGH and HPXGH motifs. Figure S6D shows a hypothetical structure with a mercury ion bound between His1 and His3 and a second ion bound to dansyl and to a terminal ligand, for example, a chloride ion released from the dissociation of HgCl_2_ (the salt used in this work). The arrangement dansyl–Hg–Cl, different from all the other complexes described so far, could explain the absence of the turn-on phase. However, we cannot exclude other possible binding modes.

Finally, Figure S7 (Supplementary Materials) shows how peptide AcH3w can bind two mercury ions even in the absence of the dansyl group, thus explaining the two different slopes in the plot of the total fluorescence as function of the Hg(II) concentration (Figure S5B).

## 4. Conclusions

For this work, we demonstrated that peptide dH3w is a very useful sensor for zinc and mercury and, potentially, the prototype for the development of a new family of mercury fluorescent sensors. Unfortunately, its unusually complicated behavior and the impossibility to solve the structures of its complexes with Zn(II) and Hg(II) make it difficult to rationally design improved variants. Here, we reported a molecular dissection of the peptide that helps to understand the molecular basis of its features. Our analysis clearly demonstrates that all the histidine residues are essential for high-affinity zinc binding. However, His3 gives a contribution slightly lower than His1 and His5. At the moment, it is still unclear if His3 is directly bound to zinc or if it performs a structural role stabilizing the turn-like structure that, as also suggested by the in silico analysis, might be necessary for the optimal positioning of His1 and His5. In the case of Hg(II), the possibility of multiple binding events makes the analysis even more complicated. As for the first binding event, our data demonstrate that two histidine residues are sufficient as long as His5 is present. This finding allows us to conclude that the motifs HPXGH and HGH (both including His5) have a considerably higher affinity for Hg(II) than the motif HPH. Moreover, the motif HPXGH (present in dH3w(H3A)) is slightly more efficient than the motif HGH (present in dH3w(H1A)); therefore, also in the case of mercury binding, the turn-like structure seems particularly favorable. As for the second binding event, which causes the turn-on response, the only plausible structure is characterized by a mercury ion bound to the dansyl group, attached at the N-terminus, and His5, thus indicating once again the importance of the turn-like structure.

Overall, our data point to His3 as a residue which could be modified to tune the features of the sensor without significant loss of the metal binding ability, thus paving the way to the development of new improved sensors.

## Figures and Tables

**Table 1:** λ_max_ values of dH3w and its variants.

		λ_max_ (nm)	
Peptide	Unbound	Zn(II) Complex	Hg(II) Complex
dH3w	560	515	507
dH3w(H1A)	560	534	507
dH3w(H3A)	560	528	515
dH3w(H5A)	560	500	515
dH3w(W6A)	560	524	510
AcH3w	359	359	350

**Table 2:** Binding constant (K_b_) and stoichiometry (n) for the Zn/peptide complexes.

Peptide	K_b_ (M^−1^)	n (peptide/zinc)
dH3w	(5.9 ± 2.2) × 10^5^	0.5 ^a^
dH3w(H3A)	(2.6 ± 0.2) × 10^4^	0.5 ^b^
dH3w(W6A)	(4.6 ± 0.2) × 10^5^	0.5 ^a^
AcH3w	(2.9 ± 0.1) × 10^5^	0.5 ^b^

^a^ Values determined through the fitting procedure. ^b^ Preset values.

**Table 3:** Energy values of the Monte Carlo minimized models of the (dH3w)_2_Zn(II) complex.

	Protonation State of His1, His3, and His5 ^a^							
Octahedral complexes	Nδ/Nδ/Nδ	Nδ/Nε/Nδ	Nδ/Nδ/Nε	Nδ/Nε/Nε	Nε/Nδ/Nδ	Nε/Nε/Nδ	Nε/Nδ/Nε	Nε/Nε/Nε
Energy (kJ/mol)	−1205.8	−1295.4	−1315.4	−1338.0	−1297.5	−1324.2	−1399.1	−1377.4
Tetrahedral complexes	Nδ/Nδ/Nδ	Nδ/Nε/Nδ	Nδ/Nδ/Nε	Nδ/Nε/Nε	Nε/Nδ/Nδ	Nε/Nε/Nδ	Nε/Nδ/Nε	Nε/Nε/Nε
Energy (kJ/mol)	−1486.6	−1478.2	−1511.7	−1510.0	−1528.0	−1526.7	−1513.4	−1512.9

^a^ Nδ and Nε indicate, respectively, histidine residues with δ1 and ε2 nitrogen atoms available for coordination. δ1 and ε2 nitrogen atoms actually bound to zinc are underlined.

## Supplementary Materials

The following are available online at https://www.mdpi.com/1424-8220/20/3/598/s1, **Figure S1.** Schematic structure of the possible dimeric Zn(II) complexes of dH3w and its variants. Only symmetric dimers are shown. The two peptide units are shown in black and blue. Zn(II) is shown as a light gray sphere, whereas the dansyl group is shown as a green “d”. **Figure S2.** Positioning of the dansyl group in the Monte Carlo minimized models of the complex Zn(dH3w)_2_. The models shown in the corresponding panels of Figure 4 are shown in an orientation chosen to highlight the molecular contacts of the dansyl group with the carbon atoms in magenta. Colors and stile are the same as in Figure 4, except that the residues which contribute to shield the dansyl group from the solvent are shown as van der Waals spheres. **Figure S3.** Fluxional model for the (dH3w)_2_Zn complex. Symbols and colors as in Figure S1. Other possible interconversion pathways (e.g., involving peptides with a single histidine coordinated to zinc) are not shown. **Figure S4.** Close up of the plots shown in Figure 5B,D,F. The normalized area of the fluorescence emission is shown as function of the Hg(II) concentration for (**A**) dH3w(H1A), (**B**) dH3w(H3A), and (**C**) dH3w(H5A) (filled circles). For comparison, the normalized area of the fluorescence emission of dH3w is also reported in each panel (void circles). Spectra were registered after excitation at 340 nm (the absorption maximum of the dansyl group) in 20 mM MOPS buffer, pH 7 at 25 °C. **Figure S5.** Close up of the plots shown in Figure 6B.D. The normalized area of the fluorescence emission is shown as function of the Hg(II) concentration for (**A**) dH3w(W6A) and (**B**) AcH3w (filled circles). (**B**) The normalized area of the fluorescence emission of dH3w is shown for comparison (void circles). Spectra of dH3w(W6A) were registered after excitation at 340 nm (the absorption maximum of the dansyl group). Spectra of AcH3w were registered after excitation at 295 nm. **Figure S6.** Schematic structure of the possible complexes of dH3w and its variants with Hg(II). The dansyl group is shown as a gray “d” in the complexes responsible for turn-off phases without changes in the λ_max_ values; a cyan “d” in the complexes responsible for turn-off phases with changes in the λ_max_ values; and a black “d” on a cyan background in the complexes responsible for the turn-on phase. Hg(II) is shown as a dark gray sphere. White pentagons represent the imidazole groups of the histidine side chain. “X” can be a chloride ion (released from the dissociation of HgCl_2_, the salt used in all the titrations) or the histidine side chain of another peptide molecule. The dashed line indicates a possible weak interaction between His3 and mercury in the (Hg)dH3w complex. **Figure S7.** Schematic structure of the possible complexes of AcH3w with Hg(II). “acH” indicates the acetylated histidine residue at the N-terminus. Other symbols as in Figure S6.
