# Supplementary material for: Molecular Dissection of dH3w, A Fluorescent Peptidyl Sensor for Zinc and Mercury
Source: Sensors (Basel). 2020 Jan 21;20(3):598. doi: 10.3390/s20030598 (PMC7038057; doi:10.3390/s20030598)
Supplement: Supplementary file 1 [file sensors-20-00598-s001.pdf]

Supplementary Material

Molecular Dissection of dH3w, A Fluorescent Peptidyl Sensor for Zinc and Mercury

Marialuisa Siepi [1,§], Rosario Oliva [2,3,§], Filomena Battista [3], Luigi Petraccone [3], Pompea Del Vecchio [3], Viviana Izzo [4], Fabrizio Dal Piaz [4], Rachele Isticato [1], Eugenio Notomista [1,*,†] and Giuliana Donadio [4,*,†]

1 Department of Biology, University of Naples Federico II, Via Cintia, 80126 Naples, Italy; marialuisa.siepi@unina.it (M.S.); rachele.isticato@unina.it (R.I.)

2 Physical Chemistry I, TU Dortmund University, Otto-Hahn-Str. 4a, 44227 Dortmund, Germany; rosario.oliva2@unina.it

3 Department of Chemical Sciences, University of Naples Federico II, Via Cintia, 80126 Naples, Italy; filomena.battista@unina.it (F.B.); luigi.petraccone@unina.it (L.P.); pompea.delvecchio@unina.it (P.D.V.)

4 Department of Medicine, Surgery and Dentistry "Scuola Medica Salernitana", University of Salerno, Via Salvador Allende, 84081 Baronissi, Italy; vizzo@unisa.it (V.I.); fdalpiaz@unisa.it (F.D.P.)

* Correspondence: notomist@unina.it (E.N.), giuliana.donadio@unina.it (G.D.)

† These authors contributed equally to this work.

§ These authors contributed equally to this work.

Received: 16 December 2019; Accepted: 17 January 2020; Published: date

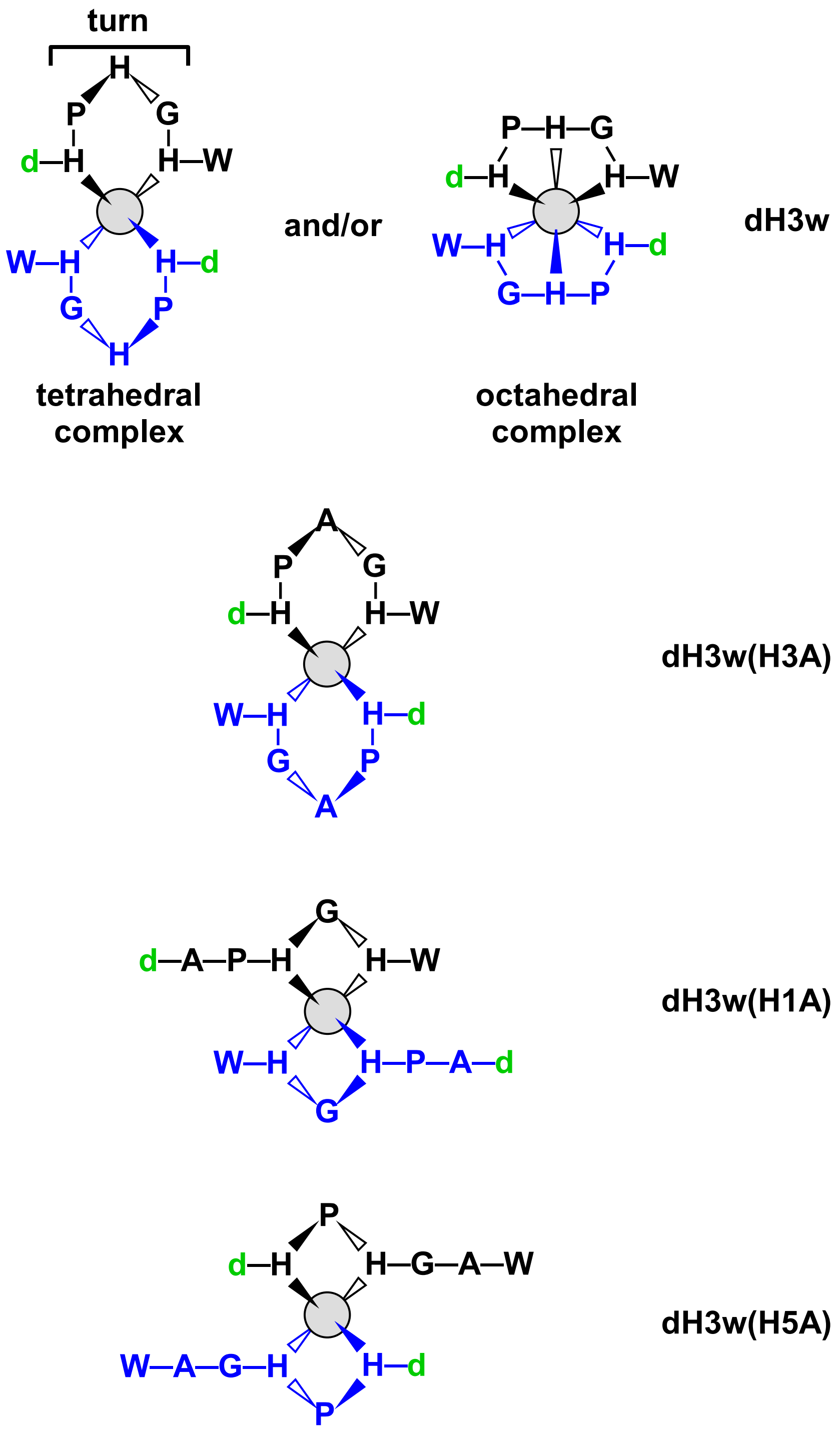

Figure S1. Schematic structure of the possible dimeric Zn(II) complexes of dH3w and its variants. Only symmetric dimers are shown. The two peptide units are shown in black and blue. Zn(II) is shown as a light gray sphere, whereas the dansyl group is shown as a green "d".

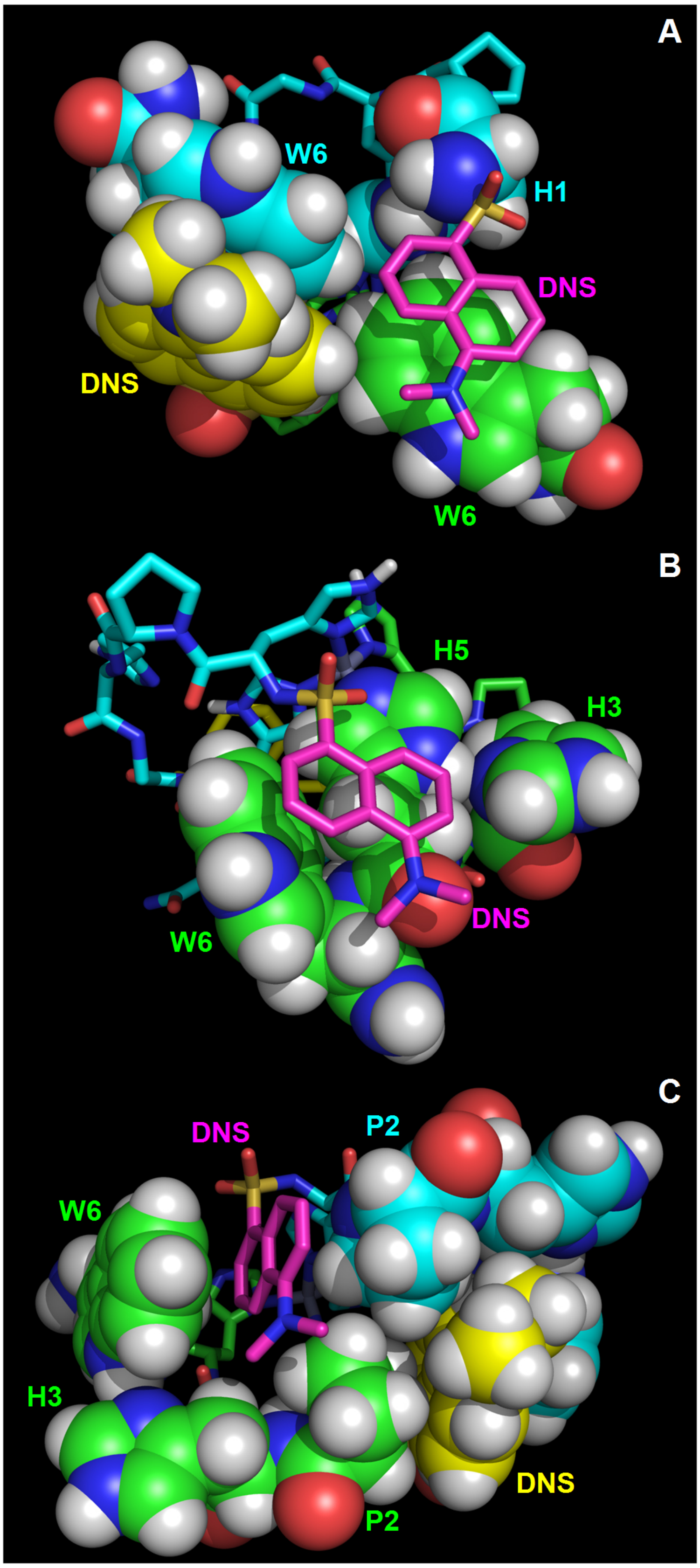

Figure S2. Positioning of the dansyl group in the Monte Carlo minimized models of the complex Zn(dH3w)$_2$. The models shown in the corresponding panels of Figure 4 are shown in an orientation chosen to highlight the molecular contacts of the dansyl group with the carbon atoms in magenta. Colors and stile are the same as in Figure 4, except that the residues which contribute to shield the dansyl group from the solvent are shown as van der Waals spheres.

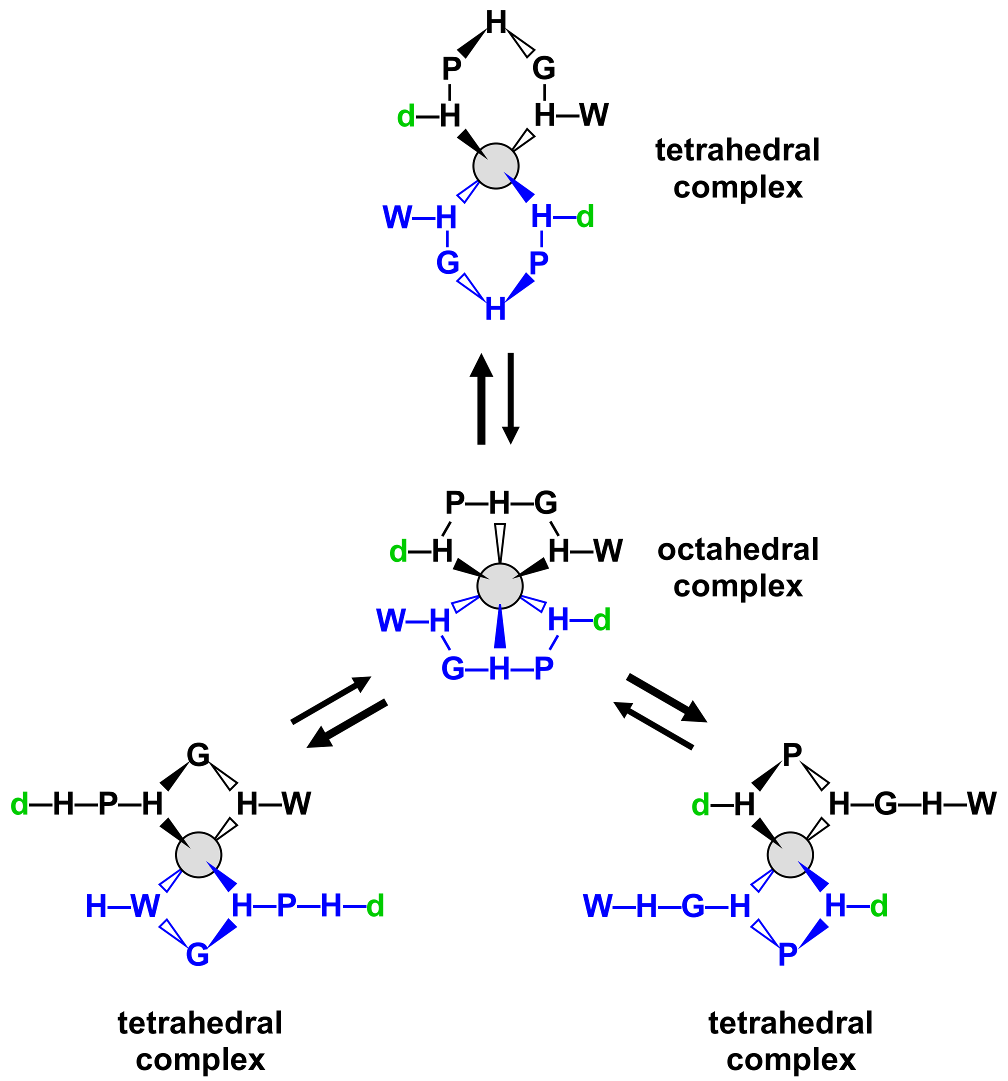

Figure S3. Fluxional model for the $(dH3w)_2Zn$ complex. Symbols and colors as in Figure S1. Other possible interconversion pathways (e.g., involving peptides with a single histidine coordinated to zinc) are not shown.

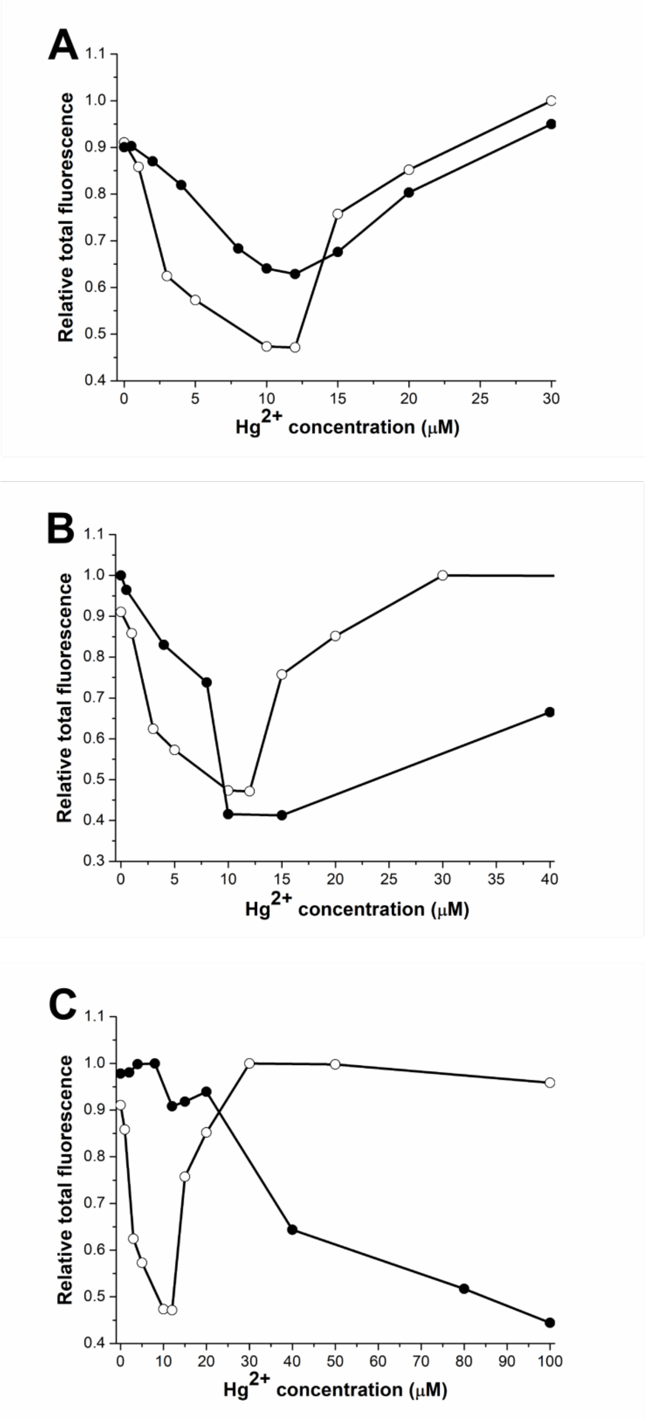

Figure S4. Close up of the plots shown in Figure 5B,D.F. The normalized area of the fluorescence emission is shown as function of the Hg(II) concentration for (**A**) dH3w(H1A), (**B**) dH3w(H3A), and (**C**) dH3w(H5A) (filled circles). For comparison, the normalized area of the fluorescence emission of dH3w is also reported in each panel (void circles). Spectra were registered after excitation at 340 nm (the absorption maximum of the dansyl group) in 20 mM MOPS buffer, pH 7 at 25 °C.

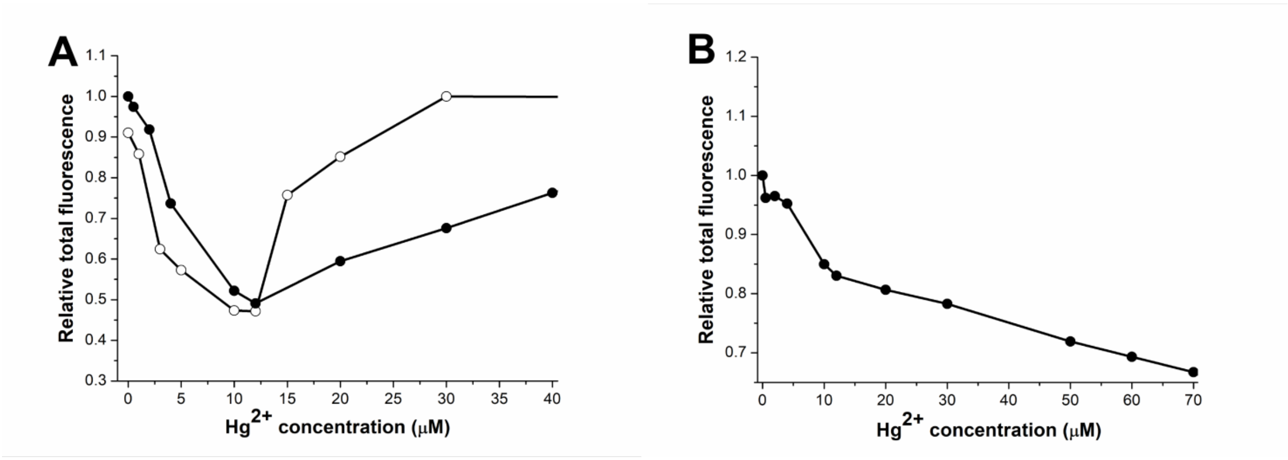

Figure S5. Close up of the plots shown in Figure 6B.D. The normalized area of the fluorescence emission is shown as function of the Hg(II) concentration for (**A**) dH3w(W6A) and (**B**) AcH3w (filled circles). (**B**) The normalized area of the fluorescence emission of dH3w is shown for comparison (void circles). Spectra of dH3w(W6A) were registered after excitation at 340 nm (the absorption maximum of the dansyl group). Spectra of AcH3w were registered after excitation at 295 nm.

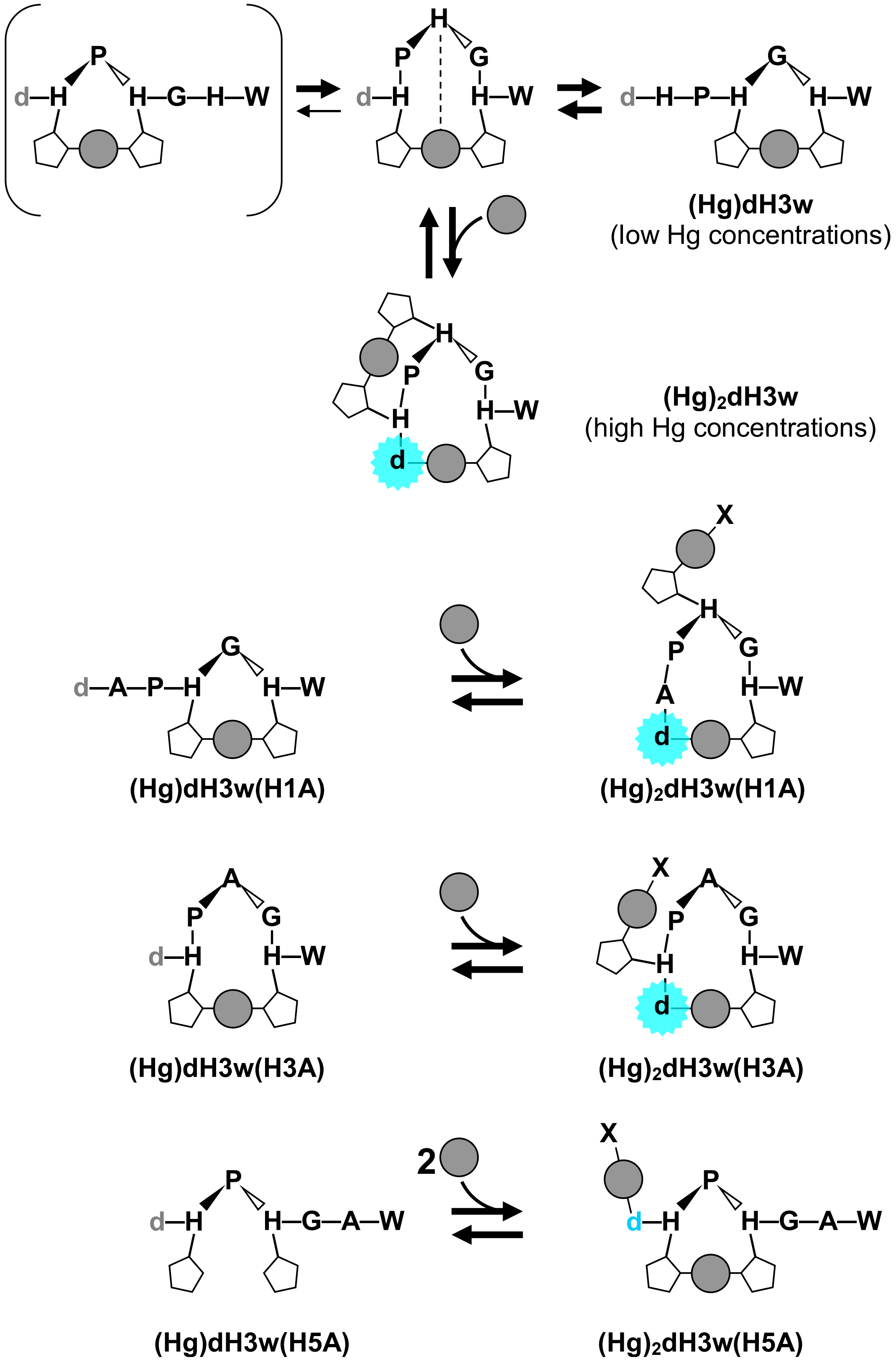

Figure S6. Schematic structure of the possible complexes of dH3w and its variants with Hg(II). The dansyl group is shown as a gray "d" in the complexes responsible for turn-off phases without changes in the λ_{max} values; a cyan "d" in the complexes responsible for turn-off phases with changes in the λ_{max} values; and a black "d" on a cyan background in the complexes responsible for the turn-on phase. Hg(II) is shown as a dark gray sphere. White pentagons represent the imidazole groups of the histidine side chain. "X" can be a chloride ion (released from the dissociation of

$HgCl_2$, the salt used in all the titrations) or the histidine side chain of another peptide molecule. The dashed line indicates a possible weak interaction between His3 and mercury in the (Hg)dH3w complex.

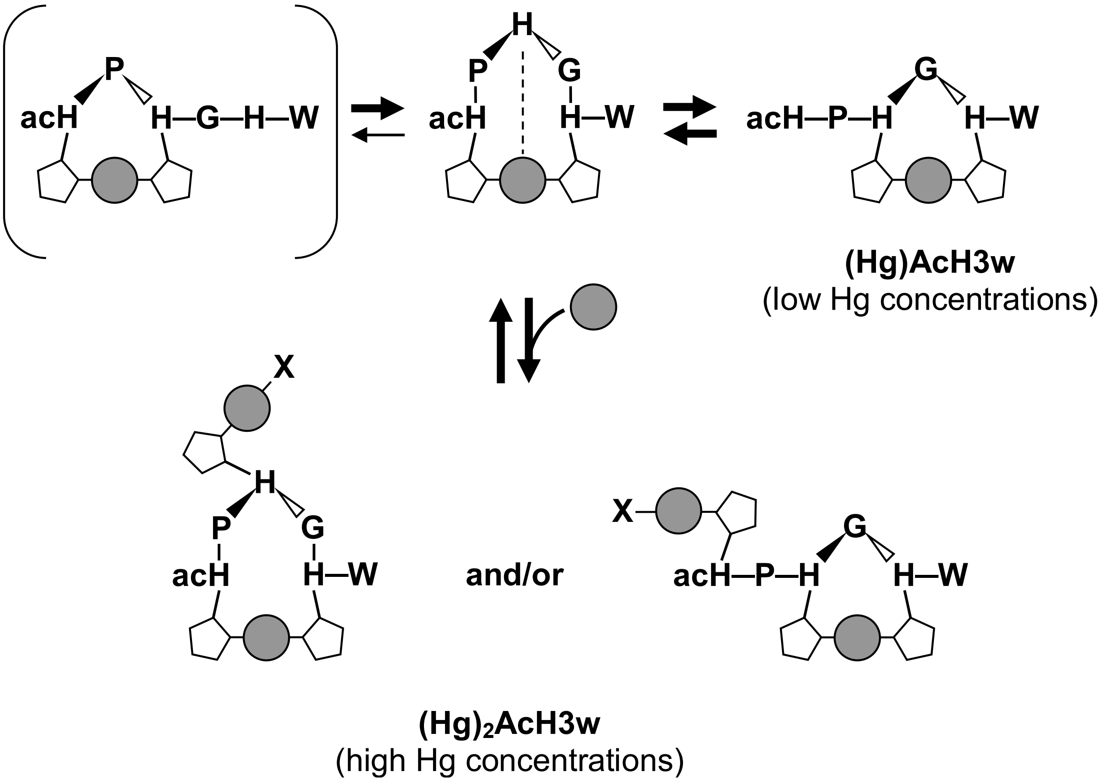

Figure S7. Schematic structure of the possible complexes of AcH3w with Hg(II). "acH" indicates the acetylated histidine residue at the N-terminus. Other symbols as in Figure S6.
